# Dyslipidemia and Its Associated Factors Among People Living With HIV on Dolutegravir-Based Antiretroviral Therapy at Debre Markos Comprehensive Specialized Hospital, Northwest Ethiopia: A Cross-Sectional Study

**DOI:** 10.1155/arat/6621097

**Published:** 2025-08-05

**Authors:** Mohammed Jemal, Adane Adugna, Mamaru Getinet, Baye Ashenef, Gelagey Baye, Desalegn Abebaw, Zigale Hibstu Teffera, Wastina Bitewlign, Anemut Tilahun, Nuredin Chura Waritu, Tabarak Malik, Enyew Fenta Mengistu

**Affiliations:** ^1^Department of Biomedical Science, School of Medicine, Debre Markos University, Debre Markos, Ethiopia; ^2^Department of Medical Laboratory Sciences, College of Health Sciences, Debre Markos University, Debre Markos, Ethiopia; ^3^Department of Biochemistry, College of Medicine and Health Science, University of Gondar, Gondar, Ethiopia; ^4^Department of Biomedical Sciences, College of Health Sciences, Debre Tabor University, Debre Tabor, Ethiopia; ^5^Department of Biomedical Sciences, School of Medicine, Wolaita Sodo University, Wolaita Sodo, Ethiopia; ^6^Division of Research & Development, Lovely Professional University, Phagwara, India; ^7^Department of Biomedical Sciences, Institute of Health, Jimma University, Jimma, Ethiopia

**Keywords:** ART, dolutegravir, dyslipidemia, high-density lipoprotein cholesterol, HIV, low-density lipoprotein cholesterol, total cholesterol, triglyceride

## Abstract

**Background:** Dyslipidemia is a prevalent public health concern among individuals living with HIV who are undergoing antiretroviral therapy (ART), and it increases the risk of cardiovascular diseases. Despite the introduction of improved medications like dolutegravir, there are limited data regarding the extent of dyslipidemia in this population. Therefore, the purpose of this study was to determine the prevalence of dyslipidemia and its associated factors among people living with HIV on dolutegravir-based ART.

**Methods:** An institutional-based cross-sectional study was carried out from December 1, 2021, to February 30, 2022. Sociodemographic, behavioral, clinical, anthropometric, and laboratory data were collected from 423 participants. Simple random sampling methods were used to recruit the participants. The collected data were entered in Epi-data Version 4.6 and analyzed via SPSS Version 26. Multivariable logistic regression was used to determine factors associated with dyslipidemia. A *p* value of < 0.05 was considered statistically significant.

**Results:** The prevalence of dyslipidemia was found to be 73.8%. Low HDL-C was the most common (61.2%), followed by elevated levels of TG (40.2%), high LDL-C (26%), and high TC (24.3%). Being female (AOR = 1.77, 95% CI: 1.04–3.02, *p*=0.034), insufficient physical activity (AOR = 1.64, 95% CI: 1–2.67, *p*=0.048), being overweight (AOR = 2.25, 95% CI: 1.07–4.75, *p*=0.033), and being obese (AOR = 2.32, 95% CI: 0.86–6.25, *p*=0.045) were significantly associated with dyslipidemia.

**Conclusion:** The prevalence of dyslipidemia among people living with HIV on a dolutegravir-based ART was high, occurring in nearly three-quarters of the participants. Being female, insufficient physical activity, and being overweight or obese were significantly associated with dyslipidemia among people living with HIV taking dolutegravir-based ART. Therefore, to avoid the disastrous effects of dyslipidemia, serum lipid profiles should be considered and evaluated in people living with HIV on a dolutegravir-based ART.

## 1. Background

Metabolic disorders are commonly found in people living with HIV (PLHIV) and occur more frequently than in healthy control groups [1, 2]. Long-term use of antiretroviral therapy (ART) has led to cardiovascular diseases (CVDs) becoming the primary cause of mortality among PLHIV [3, 4]. Dyslipidemia is the most prevalent CVD risk factor in PLHIV [5] and is responsible for approximately 50% of the overall CVD risk [6, 7]. Therefore, selecting a more lipid-friendly regimen is imperative to improve dyslipidemia and reduce CVD burdens in PLHIV [8, 9].

Nowadays, the World Health Organization (WHO) suggests dolutegravir (DTG), an integrase strand transfer inhibitor (INSTI), as a preferred first-line and second-line ARTs for all PLHIV, including those who are pregnant or of childbearing age, because of its noninferiority antiviral efficacy, high genetic barrier to resistance, and lower potential for side effects [10]. Since 2018, the Ministry of Health (MoH) in Ethiopia has recommended the use of a DTG-containing regimen as the preferred first-line treatment for adults and adolescents living with HIV [11].

PLHIV who are on ART are prone to encountering various side effects and adverse drug reactions, such as hyperglycemia and dyslipidemia [12, 13]. Recent investigations have found that ART is linked to a higher level of triglyceride (TG), total cholesterol (TC), and low-density lipoprotein cholesterol (LDL-C) and a lower level of high-density lipoprotein cholesterol (HDL-C) in the blood [14, 15]. After the introduction of combination ART, research showed that older nonnucleoside reverse transcriptase inhibitors (NNRTIs) and boosted protease inhibitors (PIs) were associated with a higher risk of dyslipidemia than more modern ARTs in these categories [16, 17]. In the past 10 years, new classes of ART such as INSTI have been developed. Compared with other antiretroviral drugs, INSTI may have a superior lipid safety profile, according to results from clinical investigations [18, 19]. In laboratory settings, INSTI inhibits endoplasmic reticulum stress, an important pathway for the dyslipidemia induced by ART [20, 21]. Nonetheless, INSTI causes oxidative stress and insulin resistance and is generally linked to a notable weight gain in certain PLHIV [22–24]. Recently, compared with people on NNRTIs such as efavirenz (EFV), people taking DTG-based regimens have a higher incidence of dyslipidemia [25]. A cross-sectional study conducted in Zambia among 100 NNRTIs, 100 PIs, and 100 DTG-treated patients showed that DTG-based ART was significantly associated with a decreased level of HDL-C [26]. This could potentially increase the risk of CVD, as HDL-C is known for its antiatherogenic effects [27].

A cross-sectional study carried out in Uganda among 341 PLHIV taking DTG revealed that the magnitude of dyslipidemia was 78.0% [28]. Two comparative cross-sectional studies in Ethiopia reported different prevalence rates of dyslipidemia among PLHIV taking DTG-based ART, with rates of 79.7% in Northeast Ethiopia [25] and 48.4% in southwest Ethiopia [29]. The prevalence of dyslipidemia in PLHIV on DTG-based ART is not known precisely. Hence, there is a significant gap that needs to be studied since the majority of PLHIV in Ethiopia are on DTG-based ART.

Despite this, lipid profile levels are not frequently assessed among PLHIV in Ethiopia because of financial constraints. So far as we know, there are currently no studies reporting the extent of dyslipidemia and its associated factors among PLHIV taking DTG-based ART, particularly in the study area. Therefore, the aim of this study was to assess the magnitude of dyslipidemia and its associated factors among PLHIV on DTG-based ART at Debre Markos Comprehensive Specialized Hospital (DMCSH), Northwest Ethiopia.

## 2. Materials and Methods

### 2.1. Study Area, Design, and Period

An institution-based cross-sectional study was carried out at DMCSH from December 1, 2021 to February 30, 2022 in the East Gojjam Zone of the Amhara National Regional State. The hospital serves more than 7 million individuals in the town and surrounding districts. Alongside general healthcare service, it provides interventions for HIV/AIDS including diagnosis, treatment, and monitoring. As of November 21, 2021, the hospital was delivering ART services to more than 3574 patients living with HIV/AIDS.

### 2.2. Population and Eligibility Criteria

The source populations were all PLHIV on DTG-based ART at the ART Clinic of DMCSH. We enrolled all PLHIV aged 18 years or older who had been on DTG-based ART (DTG + 3TC + TDF) for more than 6 months. Those who had diabetes mellitus, active cancer, renal disease, chronic liver disease, mental health problems, and patients who were not able to provide appropriate information were excluded.

### 2.3. Sample Size and Sampling Procedure

The sample size was calculated using a single population proportion formula with the following assumptions: 50% prevalence of dyslipidemia because of no previous study in the area, 5% margin of error, 95% confidence interval (CI), and 10% for nonresponse. The total sample became 423. At the ART clinic in the hospital, the daily patient flow was accessed from the data logbook so as to select the study subjects. The study subjects were recruited using a simple random sampling technique by lottery method.

### 2.4. Data Collection and Procedure

We collected data using a structured questionnaire modified from the WHO stepwise approach to chronic disease risk factor surveillance questions [30]. The questionnaire used in this study was pretested on 5% (*n* = 21) of the sample size for validation. The questionnaire is divided into three sections: the first focuses on face-to-face interviews and medical record reviews; the second on anthropometric and blood pressure measurements; and the third on biochemical measurements. All data were collected by nurse professionals and laboratory technologists under the close supervision of the principal investigator. The data collectors received 2 days of training on the purpose of the study, data collection procedures, and techniques for gathering information from participants.

Face-to-face interview and medical record review: Data on demographic (age, gender, residence, educational status, marital status, religion, and occupation), behavioral (alcohol intake, smoking, and physical activity), and clinical (WHO HIV stage, duration of HIV infection, ART duration, duration on DTG, history of opportunistic infections, drug adherence level, family history of diabetes mellitus, hypertension, and CVD) characteristics were collected directly from participants and from patients' medical records.

Anthropometric and blood pressure measurements: Body weight and height were measured using a digital balance with a height measurement attached to it. Weight was measured (to the nearest 100 g) by placing the weighing balance on a flat hard surface and height was measured (to the nearest 0.1 cm) while a patient was facing directly ahead. Body mass index (BMI) was determined by dividing weight in kilograms (kg) by height in meters squared (m^2^). Both the waist circumference (WC) and hip circumference (HC) were determined using a flexible inelastic tape to the nearest 0.1 cm. WC was measured over light clothing at the approximate halfway point between the lower margin of the last palpable rib and the top of the iliac crest after breathing out, when the lungs have reached their functional residual capacity, with the subject standing and weight evenly distributed across the feet. HC was also measured over light clothing at the level of the greater trochanters, with the subject standing and both feet together. Blood pressure (BP) was measured using an automated sphygmomanometer with an appropriately sized cuff, while the participant was seated. Participants were instructed to rest for at least 5 min prior to measurement. If they had consumed caffeinated beverages, a rest period of 30 min was observed before proceeding with the measurement. The participant's final BP was calculated by averaging three measurements that were taken at 5 min intervals.

Biochemical measurements: Approximately 9 mL of fasting venous blood were collected from each participant by laboratory technologists. Fasting status was confirmed with participants prior to blood sample collection. If fasting had not been observed, the blood draw was rescheduled accordingly. The process of blood sample collection took place through an aseptic/sterile technique. Blood samples in vacutainer tubes were clearly labeled and transported immediately to the laboratory for analysis in a cooler box. The samples for TC, HDL-C, LDL-C, and TG were collected in green-topped containers (lithium heparin). Blood samples were centrifuged at 10,000 relative centrifugal force (RCF) for 10 min to separate serum from whole blood. Lipid profiles (TC, TG, LDL-C, and HDL-C) were analyzed using the Roche Cobas C111 clinical chemistry analyzer (Roche Diagnostics, Basel, Switzerland) following the manufacturer's protocol. The analyzer uses enzymatic colorimetric assays to determine lipid profiles. Test results were evaluated and classified according to the definition. In addition, CD4 count (cells/μL) samples were collected in ethylenediaminetetraacetic acid (EDTA) containers and assayed using a Becton Dickson flow cytometer. Furthermore, viral load (copies/mL) samples were collected in EDTA containers and analyzed using a COBAS® Ampliprep/COBAS® TaqMan 96 PCR analyzer.

### 2.5. Operational Definitions

Dyslipidemia was defined on the basis of the National Cholesterol Education Program Adult Treatment Panel III guidelines as LDL-C ≥ 130 mg/dL, TC ≥ 200 mg/dL, TG ≥ 150 mg/dL, and HDL-C < 40 mg/dL for men and < 50 mg/dL for women, occurring in isolation or in combination [31].

BMI was classified as underweight (< 18.5 kg/m^2^), normal weight (18.5–24.9 kg/m^2^), overweight (25–29.9 kg/m^2^), and obese (≥ 30 kg/m^2^) [32].

Hypertension was defined as a systolic blood pressure ≥ 140 mmHg and/or diastolic blood pressure ≥ 90 mmHg [33].

WC: cutoff point for females > 80 and > 94 cm for males [34].

Waist-to-hip ratio: cutoff point for females ≥ 0.85 and ≥ 0.9 for males [34].

Fasting is defined as no caloric intake for at least 8 h [35].

Regarding physical activity: Vigorous-intensity activities involve substantial physical effort and lead to significant increases in breathing or heart rate for at least 10 min continuously (e.g., running, carrying or lifting heavy loads, digging, or construction work). Moderate-intensity activities are activities that require a moderate physical effort and cause small increases in breathing or heart rate for at least 10 min continuously [36].

Sufficient physical exercise: Adults should engage in a minimum of 150–300 min of moderate-intensity aerobic physical activity, or 75–150 min of vigorous-intensity aerobic physical activity, or an equivalent combination of moderate-intensity and vigorous-intensity physical activities per week and otherwise insufficient [37].

Smoking status was defined as “smoker” for participants who had smoked at least one cigarette within the last 1 year [38].

Alcohol drinking status was defined as “alcohol drinker” for participants who consumed any type of alcoholic beverage more than once per week in the past year regardless of the amount [39, 40].

### 2.6. Data Processing and Analysis

The gathered data were cleaned, coded, and entered into Epi-data Version 4.6 before being exported to the Statistical Package for Social Science (SPSS) version 26 for analysis. Descriptive statistics was performed, with results displayed in tables and figures. Categorical variables are reported as counts and percentages, and they are analyzed using the chi-square test to identify differences between the groups. Continuous variables are presented as mean ± standard deviation (SD) if normally distributed, or as median with interquartile range (IQR) if skewed. A binary logistic regression model was employed to identify the independent variables associated with dyslipidemia. Variables with *p* values < 0.25 from the bivariable logistic regression analysis were subsequently included in a multivariable logistic regression model to account for potential confounding factors. Both crude and adjusted odds ratios with 95% CI are reported, and a *p* value of < 0.05 is considered statistically significant.

## 3. Results

### 3.1. Sociodemographic and Behavioral Characteristics of the Study Participants

A total of 423 PLHIV taking DTG-based ART participated in this study. The majority of the study subjects were female (73.3%), and the median age was 41 years (IQR: 37, 49). Among the participants, 51.8% lived in rural areas, 63.8% were married, 37.6 were unable to read and write, 35.7 were farmers, and 62.4% reported their monthly income of < 4000 ETB. In addition, 42.1% of participants had insufficient physical activity, 5% had a history of smoking, and 13% reported alcohol consumption (see Table 1).

### 3.2. Anthropometric and Clinical Characteristics

Almost half of the participants in the study (49.6%) had been living with HIV for more than 7 years; 60.3% had been using a DTG-based regimen for at least 2 years; 4.3% had a nonsuppressed viral load; and 19.9% were classified as WHO clinical staging class II. In addition, 43 (10.2%) patients were obese; 22.9% had raised WC, 8.5% had raised blood pressure, and 6.1% had a family history of CVD (see Table 2).

### 3.3. Prevalence of Dyslipidemia Among the Study Participants

**Figure 1:**
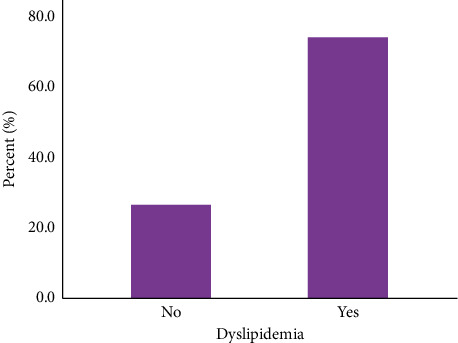
Prevalence of dyslipidemia among PLHIV taking DTG-based ART at DMCSH.

Out of the total participants, 312 individuals exhibited at least one lipid abnormality, resulting in an overall prevalence of dyslipidemia of 73.8%, as illustrated in Figure 1. The most prevalent condition was low HDL-C, occurring in 61.2% of participants, followed by elevated levels of TG at 40.2%, high LDL-C at 26%, and high TC at 24.3% (Table 3).

### 3.4. Factors Associated With Dyslipidemia Among the Study Participants

In the bivariable logistic regression, age, sex, residence, income, physical activity, alcohol intake, duration of HIV, duration on ART, duration on DTG-based ART, WHO clinical stage, CD4 T-cell count, family history of hypertension, BMI, blood pressure, and WC had *p* ≤ 0.25. After multivariable logistic regression, female sex (AOR = 1.77, 95% CI: 1.04–3.02, *p*=0.034), insufficient physical activity (AOR = 1.64, 95% CI: 1–2.67, *p*=0.048), being overweight (AOR = 2.25, 95% CI: 1.07–4.75, *p*=0.033), and being obese (AOR = 2.32, 95% CI: 0.86–6.25, *p*=0.045) were found to be significantly associated with dyslipidemia (Table 4).

## 4. Discussion

This study aimed to assess the prevalence of dyslipidemia and its associated factors in PLHIV who are undergoing DTG-based ART. The prevalence of dyslipidemia in this group was found to be 73.8%. The most common condition was low HDL-C (61.2%), followed by high TG (40.2%), high LDL-C (26%), and high TC (24.3%). In addition, the study identified gender, physical activity, and BMI as factors significantly associated with dyslipidemia among PLHIV taking DTG-based ART.

The prevalence of dyslipidemia in our study was lower than that reported in a cross-sectional study conducted in Uganda in 2022 (78%) [28]. The prevalence was also lower than that reported in a related study carried out in Northeast Ethiopia (79.7%) [25]. However, the prevalence in this study was higher than that reported in Southwest Ethiopia (48.4%) [29]. The observed differences could be related to variations in the ART exposure history of the participants (ART-naïve for the study from Northeast Ethiopia versus the ART experienced in our study), sample size (small sample size for the studies from Northeast and Southwest Ethiopia versus the large sample size used in the present study), differences in the state of sample collection (fasting in our study versus random in a study from Southwest Ethiopia), and sociodemographic and behavioral characteristic variability, which might have contributed to these incomparable findings.

Low HDL-c was the most common lipid abnormality, with a prevalence rate of 61.2% in the current study. This finding is supported by studies conducted in Uganda (72.1%) [28], Zambia (59%) [26], and Northeast Ethiopia (64.1%) [25]. However, in comparative clinical trials, DTG demonstrated a neutral effect on lipid levels (HDL-C, LDL-C, and TC) when combined with NRTIs at 48 weeks [41]. Similarly, a study carried out in Italy indicated that transitioning patients to DTG-based ART improved lipid profiles after 48 weeks of treatment [42]. The discrepancy in results could be attributed to variations in racial demographics, as earlier studies (a comparative clinical trial and a study from Italy) primarily involved white participants, whereas the current and other studies from Uganda, Zambia, and Northeast Ethiopia exclusively included black Africans. This difference in race may suggest that genetic and lifestyle factors such as diet could contribute to the observed variations. Therefore, race-specific data on the impact of the DTG-based ART on lipid profile are needed. Furthermore, the markedly elevated levels of low HDL-C observed may be attributed to the fact that most participants in our study, as well as those in the Ugandan study, were ART-experienced. Evidence suggests that PLHIV who switch from EFV-based to DTG-based ART often exhibit a high prevalence of low HDL-C [43]. This is because EFV has been associated with increased HDL-C levels [44], whereas DTG is largely lipid-neutral and does not promote HDL-C elevation [41]. As a result, switching to DTG may lead to a relative decline or stagnation in HDL-C levels. This, combined with persistent HIV-related inflammation and potential DTG-associated weight gain, likely contributes to the high prevalence of low HDL-C in this population.

Female sex, insufficient physical activity, and being overweight or obese were significantly associated with an increased risk of dyslipidemia among PLHIV on the DTG-based ART. Females were 1.77 times more likely to have dyslipidemia than males were, which is supported by studies done in Uganda [28], Ethiopia [45], and Eritrea [46]. Gender disparities are partially attributed to the significant alterations in lipid profiles observed in women compared with those in men, which are influenced by intricate hormonal fluctuations during their lifespan, particularly those associated with pregnancy and menopause [47].

In addition, insufficient physical activity was significantly associated with dyslipidemia. Participants with insufficient physical activity were 1.64 times more likely to develop dyslipidemia than those who were engaged in the recommended levels of physical activity. This is similar to studies done in Uganda [48] and USA [49]. This association might be explained by the effect of adequate physical activity on burning more energy, which helps prevent the buildup of fat and weight gain [50]. In addition, physical activity reduces metabolic risk by improving physiological parameters such as systemic inflammation, insulin resistance, blood lipid profile, HIV-associated lipodystrophy, and WC [51, 52]. Thus, the risk of dyslipidemia is increased by insufficient physical exercise.

Moreover, BMI was also significantly associated with dyslipidemia. Being overweight or obese (BMI > 25 kg/m^2^) increases the likelihood of dyslipidemia among PLHIV taking DTG-based ART. This finding is supported by studies carried out in Ethiopia [53], South Africa [54], and Cameroon [55]. This may be because as BMI increases, the levels of different lipid components also tend to increase [56]. A number of studies have found that being overweight or obese can lead to an increase in the release of free fatty acids through lipolysis, which in turn contributes to the development of high triglyceride levels. In addition, the liver increases its production of very low-density lipoprotein and triglycerides, which are potential factors in the development of dyslipidemia [57, 58].

### 4.1. Strengths and Limitations of the Study

This study is among the first to assess dyslipidemia among PLHIV taking DTG-based regimen in Ethiopia, providing a foundation for future studies. Despite this strength, the study has several limitations. Self-reported data for variables such as physical activity, alcohol consumption, and smoking status may have led participants to provide desirable responses. In addition, the study lacked data on participants' dietary habits, use of concurrent medications, and hormonal therapy, all of which could potentially confound the observed prevalence of dyslipidemia. Furthermore, because the study population was obtained solely from one institution, the findings must be interpreted with caution. Moreover, as this was a cross-sectional study, it was not possible to establish a temporal relationship between dyslipidemia and the associated covariates.

## 5. Conclusion and Recommendations

From this study, we can conclude that nearly three-quarters of PLHIV taking DTG-based regimen developed dyslipidemia. These findings indicate that many people are at an increased risk of developing complications associated with dyslipidemia, such as CVD. Therefore, to avoid the disastrous effects of dyslipidemia, serum lipid profiles should be considered and evaluated in PLHIV on a DTG-based ART. Being female, having insufficient physical activity, and being overweight or obese were significantly associated with dyslipidemia among PLHIV on DTG-based ART. Therefore, it is critical to promote intervention techniques aimed at increasing physical activity and reducing body weight among PLHIV who are taking DTG-based ART. Future research should consider conducting multicenter prospective cohort studies to establish causal relationships and incorporate dietary assessments to better understand contributing factors.

## Figures and Tables

**Table 1:** Sociodemographic and behavioral characteristics of the study participants.

Variables	Category	Frequency (%)
Age^∗^ (year)		41 (37, 49)

Sex	Male	113 (26.7)
	Female	310 (73.3)

Residence	Rural	219 (51.8)
	Urban	204 (48.2)

Marital status	Single	9 (2.1)
	Married	270 (63.8)
	Divorced	81 (19.1)
	Widow/widower	63 (14.9)

Education status	Unable to read and write	159 (37.6)
	Reading and writing only	90 (21.3)
	Primary level	84 (19.9)
	Secondary level	35 (8.3)
	College and above	55 (13.0)

Occupational status	Employed	46 (10.9)
	Farmer	151 (35.7)
	Merchant	62 (14.7)
	Daily Laborers	2.7 (6.4)
	Homemaker	65 (15.4)
	Others^#^	72 (14.7)

Income in ETB	< 4000 birr	264 (62.4)
	≥ 4000 birr	159 (37.6)

Physical activity	Insufficient	178 (42.1)
	Sufficient	245 (57.9)

Smoking	Smoker	21 (5)
	Non-smoker	402 (95)

Alcohol intake	Drinker	55 (13)
	Nondrinker	368 (87)

*Note:* ETB, Ethiopian birr.

^∗^Median (interquartile range).

^#^Driver, pensioner, and students.

**Table 2:** Anthropometric and clinical characteristics of PLWH on DTG-based ART at DMCSH.

Variables	Category	Frequency (%)
Duration of HIV (year)	≤ 7	213 (50.4)
	> 7	210 (49.6)

Duration on ART (year)	≤ 5	188 (44.4)
	> 5	235 (55.6)

Duration on DTG (year)	≤ 2	168 (39.7)
	> 2	255 (60.3)

WHO clinical stages	WHO stage I	339 (80.1)
	WHO stage II	84 (19.9)

Viral load	Suppressed	405 (95.7)
	Nonsuppressed	18 (4.3)

CD4 T-cells count	< 500 cell/mm^3^	228 (53.9)
	≥ 500 cell/mm^3^	195 (46.1)

History of OIs in last 6 months	Yes	28 (6.6)
	No	395 (93.4)

Drug adherence level	Good	372 (88)
	Fair	34 (8)
	Poor	17 (4)

Family history of DM	Yes	48 (11.3)
	No	375 (88.7)

Family history of HTN	Yes	41 (9.7)
	No	382 (90.3)

Family history of CVD	Yes	26 (6.1)
	No	397 (93.9)

Body mass index	Underweight	63 (14.9)
	Normal weight	208 (49.2)
	Overweight	109 (25.8)
	Obese	43 (10.2)

Waist circumference	Normal	326 (77.1)
	Increased	97 (22.9)

Waist-to-hip ratio	Normal	276 (65.25)
	Increased	147 (34.75)

Blood pressure	Normal	387 (91.5)
	Elevated	36 (8.5)

*Note:* ART, antiretroviral therapy; DTG, dolutegravir; HTN, hypertension; OIs, opportunistic infections; CVD, cardiovascular disease.

Abbreviations: BMI, body mass index; CD4, cluster of differentiation 4; DM, diabetes mellitus; HIV, human immunodeficiency virus; WHO, World Health Organization.

**Table 3:** Dyslipidemia among PLHIV taking DTG-based ART at DMCSH.

Lipid profiles	(Mean ± SD)	Category	N (%)
Total cholesterol (mg/dL)	177.04 ± 39.78	< 200	320 (75.7)
		≥ 200	103 (24.3)

High-density lipoprotein cholesterol (mg/dL)^∗^	41.6 ± 12.98	< 40/50	259 (61.2)
		≥ 40/50	164 (38.8)

Low-density lipoprotein cholesterol (mg/dL)	112.28 ± 63.21	< 130	313 (74.0)
		≥ 130	110 (26.0)

Triglyceride (mg/dL)	134.38 ± 68.24	< 150	253 (59.8)
		≥ 150	170 (40.2)

Dyslipidemia	N/A	Yes	312 (73.8)
		No	111 (26.2)

*Note:* N, number; %, percent.

Abbreviations: N/A, not applicable; SD, standard deviation.

^∗^< 40 mg/dL for men and < 50 mg/dL for women.

**Table 4:** Factors associated with dyslipidemia among PLHIV taking DTG-based ART at DMCSH.

Variables	Category	Bivariable analysis		Multivariable analysis	
		COR (95% CI)	*p* value	AOR (95% CI)	*p* value
Age (year)	N/A	1.03 (1, 1.05)	0.055	1.03 (1, 1.06)	0.074

Sex	Male	1		1	
	Female	1.84 (1.16, 2.95)	0.010	1.77 (1.04, 3.02)	0.034^∗^

Residence	Urban	1.76 (1.13, 2.73)	0.012	1.39 (0.87, 2.24)	0.173
	Rural	1		1	

Income in ETB	< 4000	1		1	
	≥ 4000	1.36 (0.86, 2.14)	0.192	1.63 (0.84, 3.16)	0.089

Physical activity	Insufficient	1.56 (1, 2.46)	0.052	1.64 (1, 2.67)	0.048^∗^
	Sufficient	1		1	

Alcohol intake	Nondrinker	1		1	
	Drinker	1.70 (0.83, 3.51)	0.149	1.52 (0.71, 3.28)	0.285

Duration of HIV (years)	≤ 7	1		1	
	> 7	1.82 (1.17, 2.83)	0.008	1.57 (0.79, 3.09)	0.195

Duration on ART (years)	≤ 5	1		1	
	> 5	1.61 (1.04, 2.49)	0.032	1.09 (0.56, 2.14)	0.804

Duration on DTG (years)	≤ 2	1		1	
	> 2	1.35 (0.87, 2.09)	0.182	1.15 (0.71, 1.85)	0.577

WHO clinical stage	WHO stage I	1.54 (0.92, 2.58)	0.100	1.52 (0.83, 2.78)	0.174
	WHO stage II	1		1	

CD4 T-cell count (cell/mm^3^)	< 500	1		1	
	≥ 500	1.43 (0.92, 2.22)	0.113	1.36 (0.82, 2.25)	0.241

Family history of HTN	Yes	1.82 (0.78, 4.23)	0.166	1.89 (0.76, 4.70)	0.173
	No	1		1	

BMI	Underweight	1		1	
	Normal weight	1.29 (0.71, 2.37)	0.405	1.63 (0.84, 3.16)	0.147
	Overweight	1.77 (0.89, 3.54)	0.106	2.25 (1.07, 4.75)	0.033^∗^
	Obese	2.19 (0.86, 5.54)	0.099	2.32 (0.86, 6.25)	0.045^∗^

Blood pressure	Normal	1		1	
	Elevated	0.6 (0.29, 1.23)	0.163	0.61 (0.28, 1.33)	0.211

Waist circumference	Normal	1		1	
	Increased	1.61 (0.93, 2.82)	0.092	1.54 (0.85, 2.81)	0.159

*Note:* ETB, Ethiopian birr; ART, antiretroviral therapy; HTN, hypertension; DTG, dolutegravir.

Abbreviations: AOR, adjusted odd ratio; BMI, body mass index; CD4, cluster of differentiation 4; COR, crude odd ratio; HIV, human immunodeficiency virus; WHO, World Health Organization.

^∗^Statistically significant at *p* < 0.05.

## Data Availability

The data that support the findings of this study are available from the corresponding author upon reasonable request.

## Nomenclature

PLHIV: People living with HIV
DTG: Dolutegravir
BMI: Body mass index
HDL-C: High-density lipoprotein cholesterol
LDL-C: Low-density lipoprotein cholesterol
TC: Total cholesterol
TG: Triglyceride
CD4: Cluster of differentiation 4
ART: Antiretroviral therapy
CVD: Cardiovascular diseases
INSTI: Integrase strand transfer inhibitor
HIV: Human immunodeficiency virus
DMCSH: Debre Markos comprehensive specialized hospital
WC: Waist circumference
CI: Confidence interval
AOR: Adjusted odd ratio
